# Interpreting point prevalence survey data on antibiotic use: guidance for facility antimicrobial stewardship teams and national policymakers

**DOI:** 10.1093/jacamr/dlag151

**Published:** 2026-07-22

**Authors:** Vivian Twemanye, Jonathan Mayito, Suzan Nakasendwa, John Paul Waswa, Silvia Figueiredo Costa, Evangelos I Kritsotakis

**Affiliations:** Independent Researcher, Kampala, Uganda; International Centre for Antimicrobial Resistance Solutions (ICARS), Copenhagen, Denmark; Infectious Diseases Institute, Makerere University, Kampala, Uganda; Makerere University, School of Public Health, Kampala, Uganda; Infectious Diseases Institute, Makerere University, Kampala, Uganda; Department of Infectious and Parasitic Diseases, University of Sao Paulo, São Paulo, Brazil; Laboratory of Biostatistics, School of Medicine, University of Crete, Heraklion, Greece

## Abstract

Point Prevalence Surveys (PPS) are widely used to describe antibiotic use in hospitals to inform antimicrobial stewardship programmes and national policies on Antimicrobial Resistance (AMR). While PPS offer valuable insight into patterns of antibiotic exposure and prescribing practices, their metrics are often interpreted beyond their technical design. This guidance provides a practical framework for interpreting PPS data appropriately and translating them into facility-level actions and policy learning. PPS provides a cross-sectional snapshot of antibiotic exposure and prescribing practice indicators (e.g. indication, route, documentation, guideline compliance and AWaRe classification). While these parameters support quality improvement, prevalence of antibiotic use is not a proxy for antibiotic consumption or stewardship impact because it reflects both initiation and duration of therapy and is influenced by factors like case-mix. Misinterpretation can lead to policy misalignment, inappropriate benchmarking and counterproductive performance targets. We propose a structured interpretation framework to enable stewardship learning leading to corrective actions rather than performance scoring.

## Introduction

Measuring antimicrobial consumption and use generates evidence for targeted and effective interventions critical to antimicrobial stewardship (AMS) programmes that deter antimicrobial resistance.^1^ The World Health Organisation (WHO) and other international agencies have provided guidance on the standard metrics, methods and tools for regular surveys on antibiotic use curated to limited resource settings.^2,3^ Practical guidance on interpreting survey results is crucial for methodological clarity, AMS intervention design, and ensuring policy validity.

Point Prevalence Surveys (PPS) are a valid and reliable method for monitoring antibiotic use in hospitals, particularly in settings where routine use data is limited, providing a snapshot and insight into prescribing patterns and quality indicators.^2^ The adaptability of PPS makes them a good entry point for understanding hospital antibiotic use.^4,5^ As PPS implementation becomes institutionalized and repeated over time, these data are increasingly used beyond facility AMS teams, including for national reporting, benchmarking and accountability.^6^ PPS outputs are being incorporated into national reporting, benchmarking and accountability frameworks; hence, their interpretation can influence stewardship priorities, resource allocation, and performance assessment.

Despite the growing volume and policy relevance of PPS outputs, there is limited practical guidance on how these metrics should be interpreted and translated into action.^7^ For instance, in the absence of an interpretation framework, PPS prevalence, a cross-sectional measure of antibiotic exposure, is often interpreted as an indicator of ‘high’ or ‘low’ antibiotic use, and by extension as a proxy for prescribing behaviour, antimicrobial consumption, or stewardship performance.^8^ Repeated PPS are sometimes described as longitudinal and shift interpretation from serial cross-sectional description to trend and impact inference, encouraging causal conclusions that PPS is not designed to support.^9,10^ This mismatch between what PPS measures and what is inferred from it risks misdirecting stewardship priorities and misinforming policy decisions.

PPS findings highlight deviations in the patterns of antibiotic prescribing behaviour that guide stewardship enquiry. However, it does not, on its own, explain why those patterns occur or which interventions are most appropriate. Effective stewardship therefore depends on interpreting PPS findings alongside clinical context, case-mix, diagnostic capacity, and complementary sources of antimicrobial use and health system data.

In this article, we provide guidance on interpreting PPS outputs appropriately and propose a structured framework to support the use of PPS data for facility-level stewardship learning and national-level system strengthening. We argue that PPS should be viewed as a diagnostic and learning tool that guides stewardship questions, informs root-cause analysis, and supports contextually appropriate interventions. We also discuss how repeated PPS can contribute to stewardship improvement while recognizing the methodological limitations of using cross-sectional surveys to infer longitudinal trends.

### The technical foundations of PPS

PPS aims to describe patterns and quality indicators of antibiotic prescribing. The indicators of consumption include overall prevalence of antibiotic use, average number of antibiotics per patient, and the route of administration.^11^ PPS prevalence describes the proportion of patients receiving an antibiotic at the time of survey. As a prevalence-based estimate, it reflects both the initiation of antibiotics (who was started) and the duration of therapy (who remains on treatment).^12^

The quality indicators captured by PPS include the indication for antibiotics, guideline compliance, duration of surgical prophylaxis, and use of microbiology investigations.^11^ These are useful ‘behavioural snapshots’ of prescribing practice quality, but they remain cross-sectional descriptors. Although PPS datasets are sometimes analysed using inferential methods, derived associations reflect co-occurrence at a single point in time and should not be interpreted as causal estimates or intervention effects.

Conducting PPS at shorter, regular intervals, rather than as isolated annual exercises, allows programmes to better contextualize observed changes in antibiotic use, helping to distinguish random fluctuations from emerging patterns in prescribing practice.^13^ Repeated PPS do not constitute true longitudinal surveillance,^14^ but they offer a pragmatic way to track stewardship changes over time.

## How to interpret PPS outputs at the facility level

### PPS prevalence

PPS prevalence can be interpreted as a measure of antibiotic exposure burden at a point in time, a trigger to ask where antibiotics are most used (ward/syndrome), or a trigger to explore duration-related drivers (e.g. prolonged courses, prophylaxis practices). A practical implication is on the threshold for action. A single PPS round showing elevated antibiotic prevalence in a ward should prompt investigation and targeted review rather than immediate stewardship intervention. Findings become more actionable when a similar pattern is observed across successive survey rounds or when ward-level stratification demonstrates a consistent deviation from expected prescribing patterns. Apparent increases in prevalence may simply reflect differences in case-mix, seasonal variation in admissions, or temporary operational factors. Distinguishing random variation from a persistent stewardship issue therefore requires at least one confirmatory data source, such as a repeat PPS, a targeted antibiotic use audit, or complementary pharmacy dispensing data.^15,16^

A critical limitation is that PPS prevalence disproportionately captures longer courses and longer stays and should not be used as a proxy for total antibiotic consumption. Patients with longer hospital stays are more likely to be present on the survey day (length-biased sampling).^17^ Prolonged hospitalization is often associated with more severe illness and extended antibiotic therapy; PPS estimates overrepresent these patients relative to those with shorter admissions or treatment courses. Consequently, PPS prevalence reflects the probability of observing antibiotic use at a single point in time rather than the total volume of antibiotics consumed and should not be used as a proxy for antibiotic consumption. Equally, low antibiotic prevalence should not be interpreted as an automatic indicator of AMS success. In high-acuity settings such as oncology or intensive care units, low prevalence may reflect differences in patient populations and prescribing practices, rather than effective stewardship, and benchmarking must always account for ward-level case-mix and clinical context.

Longitudinal estimates, such as Daily Defined Dose (DDDs) and Days of Therapy (DOT) are measures of cumulative antibiotic exposure and should not be derived directly from a cross-sectional PPS.^18^ PPS captures the start date of antibiotic therapy and the prescribed regimen on the survey day but does not contain information on the full duration of treatment and cannot be used to derive valid estimates of DDDs or DOT without making unsupported assumptions about treatment patterns e.g. dose adjustments.

AMS programmes often aim to change antibiotic initiation decisions (start/stop). PPS prevalence should not be interpreted to represent clinician prescribing tendency or antibiotic initiation rates.^18^ Prevalence is sensitive to duration and case-mix; therefore, stewardship improvements may occur without any change in prevalence, and vice versa.^19^ It should not be interpreted as an indication of how often clinicians prescribe antibiotics or as an indicator of stewardship success.

It is important to note that repeated PPS do not constitute true longitudinal surveillance. Repeated PPS rounds do not yield a true period prevalence estimate because each survey samples a different cross-section of inpatients and does not observe exposure over a defined interval within the same population.^14,20^ Changes between rounds may reflect variation in case-mix, admission patterns, treatment duration, or survey timing rather than cumulative antibiotic exposure over time. Repeated PPS can reflect directional shifts in prescribing patterns or documentation practices and should be interpreted as serial cross-sectional snapshots rather than measures of antibiotic exposure over a defined period. At the same time, the comparative value of repeated PPS should not be understated. Serial cross-sectional data across sites, facility levels, or regions can generate convergent information about prescribing pressure, class-specific overuse, or documentation gaps that, taken together, are valuable for global and national stewardship prioritization. The key interpretive principle is that these findings generate hypotheses that require further investigation, not trend estimates from a defined patient cohort.

### Prescribing quality

When correctly interpreted, PPS offer valuable insight into *how* antibiotics are used, examining prescribing behaviour and identifying priority targets for improvement. The survey findings highlight overused antibiotic classes, inappropriate surgical prophylaxis, or missing documentation, all of which provide clear, actionable targets for AMS teams. These indicators describe prescribing processes and identify opportunities for improvement. Importantly, they should be interpreted as signals of system performance rather than direct measures of individual clinician behaviour.

Interpretation should focus on the questions raised by each indicator. For example, a high Watch or Reserve proportion should prompt review of syndrome complexity, diagnostic capacity, access to antibiotic availability, and guideline content before concluding that broad-spectrum antibiotics are overused.^21,22^ Similarly, low guideline adherence may reflect outdated or impractical guidelines rather than inappropriate prescribing, while poor documentation of indication or stop/review dates often signals weaknesses in documentation systems and clinical workflows rather than irrational prescribing. Prolonged surgical prophylaxis should direct attention to perioperative protocols and accountability systems.

When conducted at regular intervals, repeated PPS can indicate directional shifts in these quality indicators. Such trends are suggestive rather than conclusive, since each round samples a different cross-section of patients, but consistent improvement or deterioration across rounds strengthens the stewardship signal.

**Figure 1.**
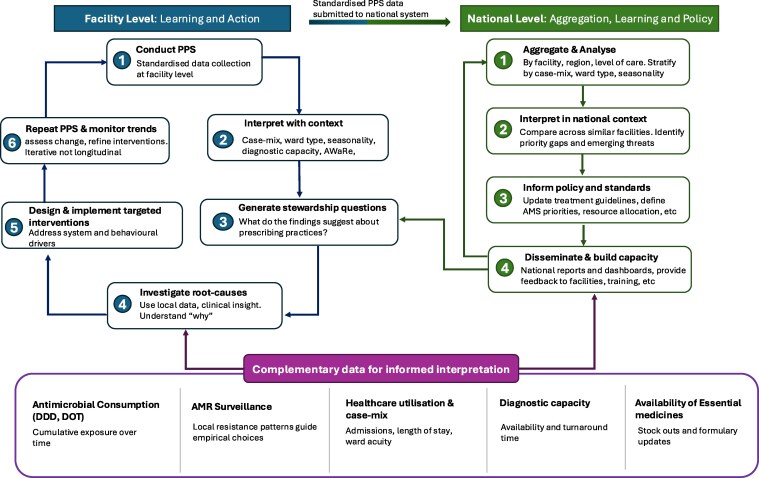
PPS as a diagnostic and learning tool within the national and facility AMS programme.

PPS data enable conservative benchmarking between hospitals. Comparisons are useful when stratified appropriately (ward type, health service level), and interpreted alongside facility context, diagnostics capacity and case-mix.^23^ PPS quality indicators also provide a rapid feedback mechanism to engage clinicians in stewardship discussions. When conducted at shorter intervals and at regular intervals, repeated PPS can suggest an upward or downward change in antibiotic use patterns, though they remain limited in their ability to distinguish random fluctuation from true behavioural change. Figure 1 summarizes the proposed framework for interpreting PPS findings and translating them into stewardship action at both facility and national levels.

## Guidance for facility AMS teams using PPS for improvement

Considering the practical guidance in the standard protocols for surveillance and data use, PPS should be operationalized as a learning tool for quality improvement.^2^ We recommend an interpretation framework for PPS outputs (Table 1).

**Table 1.** Recommended PPS interpretation framework for facility AMS

PPS indicator	What does it mean	What it does NOT mean	What to check next
Prevalence of antibiotic use	Exposure burden at a point in time	Prescribing behaviour, consumption, or AMS impact	Duration drivers, case-mix, length of stay
High Watch/Reserve (AWaRe) proportion	Escalation of treatment due to progression of illness, broader spectrum use	Resistance burden	Microbiology access and utilisation, guideline content, sepsis case-mix
Poor guideline adherence	Weak implementation	Prescriber knowledge deficit	Guideline (up to date) availability, training, feasibility, stock-outs
Prolonged surgical prophylaxis	Protocol or practice gap	High infection burden	Theatre processes, e.g. ^a^IPC, protocols, accountability systems
Missing stop/review date	Documentation system failure	Irrational prescribing	Charting tools, workflow, stewardship prompts

^a^IPC, Infection Prevention and Control.

Before acting on PPS data outputs, validate the accuracy of the denominators, identify outlier patterns that may be driven by a single unit, and assess environmental factors such as stockouts or guideline changes. In resource-limited settings, the reliability of qualitative PPS indicators is contingent on documentation infrastructure that may be inconsistent. Where patient records are paper-based, staffing is limited, or form supplies are intermittent, low rates of documented indication or stop/review dates should be interpreted as documentation gaps rather than evidence of irrational prescribing. In such contexts, PPS data should be triangulated with direct clinical observation where feasible, and interpretation should explicitly account for documentation capacity as a confounding factor.

Convert PPS outputs into stewardship questions for further investigation and root cause analysis. For instance, ‘Why is surgical prophylaxis exceeding 24 h? (visible gaps in theatre infection prevention and control and protocols, workflow?)’, ‘Why is documentation poor? (stock outs of forms, training, accountability?)’.

Translating PPS findings into prescribing change requires attention to both the content and timing of feedback. PPS data are most actionable when fed back rapidly at ward level, linked to a specific stewardship question, and reviewed by the prescribing team within the same audit cycle.^24^ In resource-limited settings where PPS rounds may be infrequent, the lag between data collection and results dissemination is a critical bottleneck: a six-month delay substantially weakens the connection between findings and practice.^25^ Facilities should aim for prompt presentation of ward-level findings where possible, using simple visual formats accessible to clinical teams without data science expertize.^26,27^

The human dimension of PPS data use deserves explicit attention. PPS findings are most likely to change prescribing behaviour when presented to clinical teams as shared diagnostic questions rather than performance judgements. Speciality-level data should be presented to relevant leads in a structured clinical forum, and stewardship teams should enter these discussions with hypotheses about system-level drivers such as stock-outs, guideline gaps, diagnostic limitations, or workflow constraints. Specialities with an inherently high propensity for antibiotic use, such as intensive care or surgery, require particular care: presenting data without contextual adjustment risks generating defensiveness rather than engagement. The goal is to position PPS data as a tool for shared problem-solving, enabling clinical teams to identify what needs to change and why, rather than functioning as a surveillance instrument measuring who is performing well or poorly.

Choose interventions linked to PPS findings. These can include revision of prophylaxis protocols, introduction of antibiotic review reminders, introduction of intravenous-to-oral switch guidance, among others.

PPS findings apply to antibiotic use in the inpatient population only. A complete picture of facility-level antibiotic use burden requires complementary methods to capture outpatient use, including admissions and bed occupancy, facility laboratory and testing volume, community-level surveys, pharmacy dispensing records, or supply-side consumption data. This scope limitation is important for stewardship teams who may otherwise treat PPS prevalence as a proxy for total antibiotic use in a health facility. Triangulate and interpret the findings with complementary facility data.^28^

## Guidance for policymakers and national antimicrobial stewardship programmes

The most consequential gap lies in how PPS results move from technical interpretation to policy use.^29^ In policy settings, PPS metrics are often treated as performance indicators and interpreted without adequate consideration of infection burden, case mix, or service delivery constraints, leading to policy actions poorly aligned with stewardship mechanisms. PPS outputs are often repurposed into accountability metrics, even though cross-sectional antibiotic exposure data cannot support simplistic targets such as ‘reduce antibiotic prevalence by X%.’

Our interpretation framework (Table 2) suggests that at the national level, PPS data should be understood in relation to national AMR surveillance systems. At the national level, PPS contributes to a broader evidence ecosystem alongside antimicrobial consumption (AMC) data, resistance patterns, and healthcare utilization indicators. PPS complements AMC metrics such as DDD and DOT by providing qualitative prescribing indicators that consumption data cannot capture, including guideline adherence and documented indication. National level interpretation should include minimum reporting standards. National PPS dashboards should at least include ward/health service level stratification, key stewardship indicators, contextual notes (infection burden, stockouts) and consumption metrics.

**Table 2.** Framework for national-level interpretation and use of PPS data

Interpretation domain	Recommended national approach	Unrecommended approach
Contextualize comparisons	Stratify analyses by facility level, ward type and service profile. Interpret findings alongside case-mix proxies and survey timing. Ensure comparability across rounds before interpreting differences.	Crude national averages or inter-facility comparisons without stratification or adjustments.
Interpret repeated PPS cautiously	Treat repeat PPS as serial cross-sectional snapshots. Assess methodological consistency (participating facilities, wards, timing, tool version) before inferring change. Seasonality should also be considered, as survey timing relative to seasonal variation in admission patterns may affect observed prevalence independently of any change in prescribing practice.	Describing repeated PPS as longitudinal prescribing surveillance or attributing observed changes to specific interventions without additional evidence.
Triangulate before policy action	Interpret PPS alongside complementary data where available (e.g. pharmacy consumption data, diagnostics capacity, resistance patterns, service expansion). Use PPS to generate hypotheses for further analysis.	Making policy decisions or setting targets based solely on PPS prevalence.
Define appropriate national use	Use PPS to identify system-level gaps (documentation, guideline alignment, spectrum distribution), prioritize stewardship support and inform training and guideline revision.	Ranking hospitals, penalising facilities, or setting crude prevalence reduction targets disconnected from clinical need and case-mix.

PPS findings should be used to diagnose health system constraints. PPS can guide the development of guidelines, priorities for training needs, Infection Prevention and Control (IPC) and diagnostic investments, and support for AMS programmes. PPS outputs, particularly prevalence, should not be used to rank hospitals or to set numerical antibiotic-use reduction targets disconnected from clinical need.

## Discussion

PPS has become institutionalized across hospitals and embedded within national reporting structures, expanding the interpretive demands on its outputs.^30^ The methodological design of PPS is critical to how the data is interpreted and used. The emphasis of this paper deliberately shifts from quantitative use metrics to qualitative prescribing indicators. Without diminishing the value of prevalence data, we bring to the foreground the richer diagnostic content that PPS generates: AWaRe classification profiles, guideline adherence rates, documented indication, and stop/review date recording. These qualitative indicators are where PPS most distinctively contributes to stewardship learning. A high Watch antibiotic proportion, for example, does not directly indicate overuse. It prompts investigation of whether escalation is driven by syndrome complexity, limited diagnostic capacity, or guideline content.

Repeating PPS rounds provides serial snapshots but does not transform the design into longitudinal prescribing surveillance.^20^ Studies describing repeated PPS as ‘longitudinal’ typically use this term operationally to denote repeated measurement over time rather than in the strict methodological sense of following the same cohort. Consequently, repeated PPS rounds cannot support longitudinal inferences about individual treatment trajectories, cumulative antibiotic exposure, or changes in prescribing within the same patients. Observed differences between survey rounds may reflect changes in case-mix, admission patterns, or seasonal variation as much as changes in prescribing practice. Our critique is therefore directed at the inferential implications of this framing rather than the studies themselves, which provide valuable descriptive evidence on prescribing patterns across successive time points.

The implications for stewardship are substantial. AMSprogrammes frequently target initiation decisions, duration optimization and review processes.^16^ Without structured interpretation, PPS outputs risk being misinterpreted as performance metrics rather than learning signals, potentially incentivizing inappropriate benchmarking or one-dimensional reduction targets.^31^

The frameworks proposed here position PPS as a tool that identifies areas requiring further investigation rather than one that confirms stewardship performance or measures intervention impact.^32^ In health facilities, PPS guides prioritization of wards, syndromes and prescribing patterns for focused audit and improvement interventions. At the policy level, aggregated PPS data illuminate system-level constraints, such as documentation practices, guideline alignment and diagnostic capacity, that contextualize antimicrobial consumption and use. In both circumstances, triangulation with complementary data sources provides actionable insights and strengthens interpretive validity.^33^

Articulation of what PPS measures and what it does not safeguards its value. When interpreted within its cross-sectional design, PPS can serve as a powerful tool for stewardship learning, guiding facility-level improvement and informing system-level priorities. Used appropriately, PPS supports action by identifying where to investigate and intervene, rather than serving as a proxy for performance or impact.
